# Intra- and inter-isolate variation of ribosomal and protein-coding genes in *Pleurotus*: implications for molecular identification and phylogeny on fungal groups

**DOI:** 10.1186/s12866-017-1046-y

**Published:** 2017-06-26

**Authors:** Xiao-Lan He, Qian Li, Wei-Hong Peng, Jie Zhou, Xue-Lian Cao, Di Wang, Zhong-Qian Huang, Wei Tan, Yu Li, Bing-Cheng Gan

**Affiliations:** 1 Soil and Fertilizer Institute, Sichuan Academy of Agricultural Sciences, Chengdu, 610066 China; 2 Jilin Agricultural University, Changchun, 130118 China; 3 Mianyang Institute of Agricultural Sciences, Mianyang, 621023 China

**Keywords:** Edible mushroom, Intra-isolate polymorphism, Specific variation

## Abstract

**Background:**

The internal transcribed spacer (ITS), RNA polymerase II second largest subunit (RPB2), and elongation factor 1-alpha (EF1α) are often used in fungal taxonomy and phylogenetic analysis. As we know, an ideal molecular marker used in molecular identification and phylogenetic studies is homogeneous within species, and interspecific variation exceeds intraspecific variation. However, during our process of performing ITS, RPB2, and EF1α sequencing on the *Pleurotus* spp., we found that intra-isolate sequence polymorphism might be present in these genes because direct sequencing of PCR products failed in some isolates. Therefore, we detected intra- and inter-isolate variation of the three genes in *Pleurotus* by polymerase chain reaction amplification and cloning in this study.

**Results:**

Results showed that intra-isolate variation of ITS was not uncommon but the polymorphic level in each isolate was relatively low in *Pleurotus*; intra-isolate variations of EF1α and RPB2 sequences were present in an unexpectedly high amount. The polymorphism level differed significantly between ITS, RPB2, and EF1α in the same individual, and the intra-isolate heterogeneity level of each gene varied between isolates within the same species. Intra-isolate and intraspecific variation of ITS in the tested isolates was less than interspecific variation, and intra-isolate and intraspecific variation of RPB2 was probably equal with interspecific divergence. Meanwhile, intra-isolate and intraspecific variation of EF1α could exceed interspecific divergence. These findings suggested that RPB2 and EF1α are not desirable barcoding candidates for *Pleurotus*. We also discussed the reason why rDNA and protein-coding genes showed variants within a single isolate in *Pleurotus*, but must be addressed in further research.

**Conclusions:**

Our study demonstrated that intra-isolate variation of ribosomal and protein-coding genes are likely widespread in fungi. This has implications for studies on fungal evolution, taxonomy, phylogenetics, and population genetics. More extensive sampling of these genes and other candidates will be required to ensure reliability as phylogenetic markers and DNA barcodes.

**Electronic supplementary material:**

The online version of this article (doi:10.1186/s12866-017-1046-y) contains supplementary material, which is available to authorized users.

## Background

The internal transcribed spacer (ITS) region is the most extensively used nuclear ribosomal gene for specific identification and phylogenetic analysis in fungal groups, and has been declared as the DNA barcode for fungi [1]. The RNA polymerase II second largest subunit (RPB2) and elongation factor 1 alpha (EF1α) have also rapidly become the most prevalent single copy protein-coding genes in fungal identification and phylogenetic reconstructions at the species level [2, 3]. Whether a molecular marker could be used to achieve authentic fungal identification depends very much on the sequence homogeneity within individual and species. However, ITS sequences could show variation within individuals, and sequence heterogeneity has been reported in many fungal groups, such as *Ganoderma*, *Sclerotium*, *Laetiporus*, *Wolfiporia*, and *Trichaptum abietinum* [4–17]. A few reports have also found that sequence polymorphism within isolates is also present in EF1α; however, no study has focused on this in depth [18]. Furthermore, intra-isolate variation of RPB2 remains unknown in fungal groups.

*Pleurotus* represents one of the most diverse groups of cultivated mushrooms, including some edible species with high commercial values and that are cultivated worldwide. Phylogenetics, taxonomy, and population genetics on this group have been widely studied using molecular markers, including ITS, RPB2, and EF1α sequences [3, 19]. During our process of performing ITS, RPB2, and EF1α sequencing on the *Pleurotus* spp., direct sequencing of PCR products failed in some isolates. Especially in EF1α sequences, most direct sequencing results showed ambiguous sequences, and double or multiple peaks were frequently observed in sequencing chromatograms. We assumed that intra-isolate sequence heterogeneities are present in these genes of *Pleurotus* spp. The presence of intra-isolate variability renders the use of ITS, RPB2, and EF1α for barcoding *Pleurotus* and other fungal groups questionable. However, in many phylogenetic studies on fungal groups, ribosomal and protein-coding genes without assessment of intra-isolate or intraspecific divergence were directly used for phylogeny reconstruction or specific identification [3, 20–22]. Under such circumstances, specific divergence might have been underestimated in some groups, while species diversity overestimated.

In this study, ITS, partial RPB2 and partial EF1α regions of different *Pleurotus* isolates were cloned and analyzed to (1) examine differences of intra-isolate polymorphism levels between genes, isolates, and species, and (2) to test the intraspecific and interspecific divergence of the 3 genes in *Pleurotus*. The present study has implications for molecular identification and phylogenetic analyses in fungal groups.

## Methods

### Collections and DNA extraction

We collected fruiting bodies of *Pleurotus*, and then performed isolations on them. The dried fruiting bodies were identified by morphological and molecular evidence. The isolated *Pleurotus* strains used in this study were deposited in the Soil and Fertilizer Institute, Sichuan Academy of Agricultural Sciences (SAAS). One hundred and 22 *Pleurotus* isolates were collected, and then ITS, RPB2, and EF1α of these isolates were amplified and sequenced. Twenty-two isolates were selected for cloning and sequence polymorphism analyses (Table 1). Fungal genomic DNAs were extracted from mycelia, according to the procedures described by Peng et al. [23]. Identifications of the fruiting bodies and corresponding isolates were compared to confirm the purification of the tested materials.

**Table 1:** List of strains used for polymorphism analysis in this study

Taxa	Isolates	Origin
*Pleurotus citrinopileatus*	p145	China: Sichuan, Chengdu
*P. citrinopileatus*	p146	China: Jilin, Changbai Mountains
*P. citrinopileatus*	p147	China: Jilin, Changbai Mountains
*P. ostreatus*	p019	China: Sichuan, Chengdu
*P. ostreatus*	p021	China: Sichuan, Chengdu
*P. ostreatus*	p024	China: Sichuan, Zhongjiang,
*P. ostreatus*	p026	China: Sichuan, Qingbaijiang,
*P. ostreatus*	p027	China: Sichuan, Qingbaijiang,
*P. ostreatus*	p028	China: Sichuan, Qingbaijiang,
*P. ostreatus*	p053	China: Sichuan, Pengxi
*P. ostreatus*	p055	China: Sichuan, Pengxi
*P. ostreatus*	p057	China: Sichuan, Zhongjiang
*P. ostreatus*	p058	China: Sichuan, Jintang
*P. ostreatus*	p069	China: Sichuan, Leshan
*P. ostreatus*	p079	China: Sichuan, Dazhou
*P. ostreatus*	p082	China: Sichuan, Zhongjiang,
*P. pulmonarius*	p073	China: Jilin, Changbai Mountains
*P. pulmonarius*	p077	China: Sichuan, Dazhou
*P. pulmonarius*	p078	China: Sichuan, Dazhou
*P. pulmonarius*	p003	China: Jilin, Changbai Mountains
*P. pulmonarius*	p038	China: Sichuan, Chengdu
*P. pulmonarius*	p041	China: Sichuan, Chengdu

### PCR, cloning, and sequencing

Universal primers ITS4 and ITS5 [24] were used for ITS amplification, primers fRPB2 5F (5′ GAYGAYMG WGATCAYTTYGG 3′) and bRPB2 7.1R (5′ CCCATRGCYTGYTTMCCCATDGC 3′) were used for RPB2 amplification, and EF116OR (5′ CCGAT CTTGTA GACGT CCTG 3′) and EF595F (5′ CGTGACTTCAT CAAGAAC ATG 3′) were used for the amplification of a part of the EF1α gene [3]. PCR conditions of ITS were as follows: 94 °C/5 min, 35 cycles of 94 °C/1 min, 55 °C/1 min, 72 °C/90 s, and a final extension step of 72 °C/10 min. PCRs of RPB2 and EF1α were performed as described by Rodriguez Estrada et al. [3]. For each isolate, PCR amplification was performed at least twice to exclude error of the polymerase. Both Taq polymerase and *pfu* polymerase were used for amplification.

Cloning was carried out with the Qiagen PCR Cloning Kit (Qiagen, Hilden, Germany) according to the manufacturer’s instructions. All PCR products were sequenced with primers M13F and M13R. At least 30 clones for each isolate and gene were sequenced to statistically substantiate results.

### Analysis of sequences

Sequences obtained in this study were deposited in GenBank. Sequences were manually checked and edited with Bioedit 7.0.9.0 [25]. Sequences from 1 gene of the same isolates and species were aligned in Mega 4.0 [26] to display polymorphisms. For the ITS region, only ITS1, 5.8S, and ITS2 were analyzed. RPB2 and EF1α sequences were trimmed for analyses. Both nucleotides and corresponding amino acid sequences were examined. Single occurrence sequence variants (singletons) in the multiple clones within a single isolate were assumed PCR or cloning errors and corrected. Nucleotide diversity (π-values) was calculated using the software DnaSP based on Nei and Li [27, 28].

## Results

### Polymorphism of ITS sequences

For *P. ostreatus*, 492 ITS sequences of 10 isolates were generated and the total length varied within species (588–591 bp). A total 32 “singletons (single occurrence sequence variants)” were supposed to be PCR errors, and 8 nucleotide substitutes were recognized as polymorphisms between these sequences (Additional file 1: Figure S1). All variants were nested in the non-coding region (5 in ITS1 and 3 in ITS2); two variants are T/C transitions, four are indels (insertions-deletions), and two are A/T transversions. In different isolates, the polymorphisms could occur in the same sites, or different sites. Only 1 variant was detected in isolate P027, while 8 were detected in the other 9 isolates (Additional file 1: Figure S1). Twenty-two ITS types were found within *P. ostreatus* (Additional file 2: Figure S2), and up to 13 types were detected within a single isolate P021 (Additional file 3: Figure S3). ITS sequence divergence within a single isolate was up to 1.35%, and intraspecific variation was also up to 1.35% in *P. ostreatus* (Table 2).

**Table 2:** Polymorphic sites, variation and nucleotide diversity (π) of ITS sequences in the tested *Pleurotus* species

Species	Nr. of clones	Nr. of polymorphic sites		Variation		Nucleotide diversity (π)	
		Intra-isolate	Intraspecies	Intra-isolate	Intraspecies	Intra-isolate	Intraspecies
*P. ostreatus*	289	8	8	1.35%	1.35%	0.0035	0.0034
*P. pulmonarius*	93	5	10	0.69%	1.30%	0.0025	0.0029
*P. ciltrinopileatus*	27	0	1	0.00%	0.20%	0.0000	0.0000

For *P. pulmonarius*, 93 ITS sequences of P038, P041, and P073 were obtained. The total length of the ITS was identical in the 3 different isolates (581 bp); 5 variable sites were recognized as singletons, and totally 10 polymorphic sites were found between these isolates (Additional file 4: Figure S4). One variant (306) was located in 5.8S, 3 in ITS1, and 6 in ITS2. Six ITS types and 5 polymorphic sites were observed in P038, 4 types and 2 variants in P041, and 4 types and 3 substitutes in P073. ITS sequence similarity was 98.70–100% within *P. pulmonarius*, and 99.31% within a single isolate (Table 2).

For *P. citrinopileatus*, 27 sequences of 3 isolates (P145, P146, and P147) were obtained. Expect for 2 “singletons”, no intra-isolate polymorphism site was observed in these isolates, while 1 inter-isolate variable site was found between them (27 sequences; Additional file 5: Figure S5). Sequence similarity within individuals was 100%, and intraspecific divergence was 0.20% (Table 2).

**Fig. 1:**
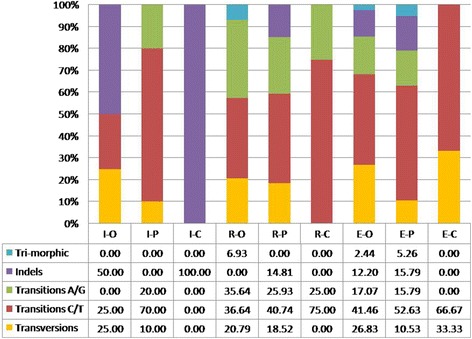
Proportion (percentage) of indels, transversions, and transitions for each gene and species. Transitions are further split into substitutions between A and G, and between C and T. I: ITS; R: RPB2; E: EF1α; O: *P. ostreatus*; P: *P. pulmonarius*; C: *P. citrinopileatus*

Of the 18 polymorphisms in the 3 *Pleurotus* species, 3 were caused by transversions, 9 by T/C transitions, 2 by A/G transitions, and 5 by indels (Fig. 1). The highest intraspecific divergence was 1.35%, and the lowest interspecific variation between the 3 tested species was 2.90%.

### Polymorphism of RPB2 sequences

For *P. ostreatus*, a total 115 sequences of P027, P028, and P053 were obtained; 11 variable sites were supposed to be singletons, and 101 variants between these sequences were considered polymorphisms (Additional file 6: Figure S6). In isolate P019, 10 RPB2 types and 20 variable sites were recovered. The 20 variable sites were composed of 12 T/C transitions, 3 A/G transitions, 5 transversions. One variant was in the intron region, and 19 in the exon region of 17 were synonymous mutations and 2 (sites 467, 687) were non-synonymous. In isolate P027, 6 sequence types and 10 variable sites (4 T/C transitions, 3 A/G transitions and 3 transversions) were observed. Among these variable bases, 4 were located in the intron region, and 6 in the exon region; 2 (486, 571) of them were non-synonymous. In isolate P028, 5 RPB2 types and 61 variable sites were found, among which 24 bases were T/C transitions, 20 were A/G transitions, and 17 were transversions. Four of these variable sites were located in the intron region, and 57 in the exon region. Only 1 variant in the exon was non-synonymous. In isolate P053, 9 sequence types and 61 variable sites were detected. Among these variable sites, 24 bases were T/C transitions, 22 were A/G transitions, 14 were transversions, and 1 was a tri-morphic site of T, C, and G. Four of these variable sites were located in the intron region, and 2 (sites 480, 828) of the 57 mutations in the exon region were non-synonymous. Sequence divergence within 1 individual as high as 6.03%, and sequence similarity within *P. ostreatus* was 93.40–100% (Table 3).

**Table 3:** Polymorphic sites, variation, and nucleotide diversity (π) of RPB2 sequences in the tested *Pleurotus* species

Species	Nr. of clones	Nr. of polymorphic sites		Variation		Nucleotide diversity (π)	
		Intra-isolate	Intraspecies	Intra-isolate	Intraspecies	Intra-isolate	Intraspecies
*P. ostreatus*	115	61	102	6.03%	6.60%	0.0294	0.0312
*P. pulmonarius*	83	20	27	1.97%	2.00%	0.0029	0.0046
*P. ciltrinopileatus*	86	8	9	0.49%	0.50%	0.0025	0.0021

For *P. pulmonarius*, 83 sequences of P003, P077, and P078 were obtained; 27 polymorphisms (Additional file 7: Figure S7) and 16 “singletons” were recognized between these sequences. Twenty-one polymorphisms were found between P003 and P077, 6 variants between P077 and P078, and 24 between P003 and P078. In isolate P003, 6 sequence types and 8 polymorphism sites were observed. Among the variable sites, we found 3 T/C transitions, 4 were A/G transitions, and 1 transversion. All polymorphism bases were in the exon region, and 2 (492, 712) were non-synonymous. Isolate P077 possessed 4 sequence types and 17 variable sites, of which 10 sites were T/C transitions, 2 were A/G transitions, 1 was a transversion, and 4 were indels. Eight of these variable sites were in the exon region and synonymous; meanwhile, 8 were in the intron region. Isolate P078 had 3 sequence types and 20 variable sites. Among these variable sites, 10 were T/C transitions, 3 were A/G transitions, 3 were transversions, and 4 were indels; furthermore, 8 sites were located in the intron region, 12 were in the exon region, and only 1 was non-synonymous. Sequence similarity within a single isolate was as low as 98.03%, and 98.00–100% within *P. pulmonarius* (Table 3).

For *P. citrinopileatus*, 86 sequences of P145, P146, and P147 were obtained; 8 polymorphisms and 5 “singletons” between these sequences were observed (Additional file 8: Figure S8). There was 1 variable site between P145 and P146, 4 between P145 and P147, and 3 between P146 and P147 (Additional file 8: Figure S8). In isolate P145, 7 sequence types and 5 variables were observed. Four of the polymorphisms were T/C transitions between, and 1 was A/G transition; 2 were in the intron region, and 3 synonymous mutation sites were in the exon region. In isolate P146, 2 sequence types and 4 variable sites (3 T/C transitions and 1 G/A transition) were detected, and 3 synonymous mutation sites were in the exon region. In isolate P147, 5 sequence types and 8 polymorphisms were found. Six polymorphisms were T/C transitions, and 2 were A/G transitions; 3 (467, 686, 948) of the 6 variations in exon region were non-synonymous. Sequence divergence within a single isolate was up to 0.49%, and 0.50% within *P. citrinopileatus* (Table 3).

Of the total 136 nucleotide substitutions in the 3 *Pleurotus* species, 26 were caused by transversions, 54 T/C by transitions, 48 by A/G transitions, 5 by indels, and 7 by tri-morphic sites. In each species, transitions from T to C were seen most frequently, followed by transitions from A to G (Fig. 1). The highest intraspecific variation was 6.60%, and the lowest interspecific divergence between the three species was also 6.60%.

### Polymorphism of EF1α Sequences

For *P. ostreatus*, 71 sequences of P019, P027, and P053 were obtained. With the exception of 23 “singletons”, 41 polymorphisms were found between these sequences (Additional file 9: Figure S9). Polymorphic sites were varied in different isolates. Thirty-one different sites were observed between P019 and P027; 39 between P019 and P053; and 19 between P027 and P053. In isolate P019, 9 sequence types and 27 variations of 14 T/C transitions, 4 G/A transitions, 4 indels, and 5 transversions were demonstrated. Seven of these variations were located in the exon region, and all of them were synonymous. In isolate P027, 5 sequence types and 5 variations of 3 T/C transitions, 1 G/A transitions and 1 transversion were found. Two polymorphisms were in the exon region, and 1 (187, 359) were non-synonymous. In isolate P053, 9 sequence types and 15 variations of 5 T/C transitions, 3 G/A transitions, 1 indel, and 6 transversions were found. Five variations were in the exon region, and only 1 (439) was non-synonymous. Sequence divergence within 1 individual was as low as 95.57%, and sequence similarity within *P. ostreatus* was 95.20–100% (Table 4).

**Table 4:** Polymorphic sites, variation, and nucleotide diversity (π) of EF1α sequences in the tested *Pleurotus* species

Species	Nr. of clones	Nr. of polymorphic sites		Variation		Nucleotide diversity (π)	
		Intra-isolate	Intraspecies	Intra-isolate	Intraspecies	Intra-isolate	Intraspecies
*P. ostreatus*	71	27	42	4.43%	4.80%	0.0163	0.0181
*P. pulmonarius*	85	12	19	2.13%	2.70%	0.0073	0.0092
*P. ciltrinopileatus*	90	2	3	0.35%	0.60%	0.0018	0.0017

For *P. pulmonarius*, a total 85 sequences (559–563 bp) of P003, P077, and P078 were obtained; 19 polymorphisms and 11 “singletons” were found (Additional file 10: Figure S10). As in *P. ostreatus*, polymorphisms between isolates in *P. pulmonarius* could also be different. There were 18 variable sites between isolates P003 and P077, 19 between P003 and P078, and only one between P077 and P078. In isolate P078, we found 12 nucleotide substitutions; furthermore, all were synonymous, and 4 (27, 45, 105, 141) were nested in the exon region, while 8 (204, 215, 217, 399, 400, 401, 402, 413) were in the intron region. Additionally, in the intron region, there was a 4 base indel (399–402). In P077, 11 polymorphisms were detected: 3 (27, 105, 141) in the exon region, and 8 (204, 215, 217, 399, 400, 401, 402, 413) in the intron region. In P003, 8 variants were found: 3 substitutions (48, 150, 548) were in the exon region, while 5 (203, 206, 211, 352, 401) were in the intron region. None of them was non-synonymous. Sequence similarity within 1 individual was as low as 97.87%, and intraspecific divergence was up to 2.70% (Table 4).

For *P. citrinopileatus*, 90 sequences of P145, P146, and P147 were obtained; 3 nucleotide substitutions and 5 “singletons” were found between these sequences (Additional file 11: Figure S11). Two of the 3 nucleotide substitutions (214: A/C; 223: T/C) were in the intron region, and 1 synonymous variation was in the exon region (189: T/C). Two variants were observed within P145, while no polymorphisms were found within P146 or P147. The lowest similarity within the same isolate was 99.65%, and sequence similarity within *P. citrinopileatus* was 99.40%–100% (Table 4).

Of the total 63 nucleotide substitutions in the 3 *Pleurotus* species, 14 were caused by transversions, 29 by transitions from T to C, 10 by transitions from A to G, 8 by indels, and 2 by tri-morphic variants. Proportions of transversions, transitions, indels for each gene and species are shown in Fig. 1. The highest intraspecific variation was up to 4.80%, but the lowest interspecific divergence between the three species was only 3.10%.

## Discussion

Intra- and inter-isolate variation of ribosomal and protein-coding genes has an appreciable effect on molecular identification and phylogenetic analysis of fungal groups. In the present study, we demonstrated that intra-isolate sequence variation is commonly present within both ribosomal and protein-coding genes of *Pleurotus*. Among the 3 tested markers, ITS showed the lowest intra-isolate and intraspecific variation level, and intra-isolate or intraspecific divergence was often less than that of interspecific divergence. However, RPB2 and EF1α sequence divergence within isolate or species was high, and could be identical or surpass the variation between species in the tested *Pleurotus* species.

ITS has been proposed as a universal DNA barcode marker for fungi [1]. As we know, a requirement of molecular markers used in taxonomic and phylogenetic studies is that all copies within the genome are constant and identical, and that intraspecific variation is lower than interspecific variation. However, intra-isolate variation in ITS regions has been reported in many fungal groups, and this variation could be observed in an unexpectedly high level in some groups. Woo et al. [16] reported 144 (19.4%) nucleotide differences between different ITS types in a single isolate of *Rhizopus microsporus*. As a general rule, ITS sequence similarities less than 90% should be considered to represent different species undoubtedly, and such a high level of variation within an individual is unbelievable. These results suggested that ITS -isolate variation level in fungal groups might be much higher than previously expected. ITS polymorphisms in *Pleurotus* have been reported in the literature. Huang et al. [29] observed a base pair indel in ITS sequences of *P. eryngii* var. *tuoliensis* (as *P. nebrodensis*), and intra-isolate variation has also been detected in *P. abolonus* [30]. In the present study, we found that the intra-isolate heterogeneity level of ITS was variable between isolates within the same species, and was different between species. On the other hand, intraspecific variation of ITS in *Pleurotus* was usually lower than variation between species in this genus. It seems that ITS could be used for specific identification in *Pleurotus*. However, it is premature to conclude that ITS is an ideal DNA barcode for *Pleurotus* due to the scarcity of samples in the present study, and care should be taken when using ITS sequences in taxonomy and phylogenetic studies in *Pleurotus* or other fungal groups for the presence of intra-isolate polymorphisms.

RPB2 has a higher specific recognition in many fungal groups, and has been used as a de facto barcode in identification and phylogenetic studies of some fungal groups, including *Pleurotus* [3, 19]. Contrary to the nuclear ribosomal genes, no intra-isolate variations were reported prior to this study in any fungal RPB2 gene sequences. In our study, RPB2 showed an unexpectedly high sequence heterogeneity level within single isolates. In the tested isolates, polymorphisms varied from 2 to 61 if “singletons” are excluded within individuals, and also differed between isolates or species. The EF1α gene was also regarded as an ideal candidate for studying the relationships of fungi. However, intra-isolate heterogeneity has also been observed in the EF1α gene of some fungal species [18] while no evidence of EF1α intra-isolate polymorphism has been found in *Pleurotus*. In this study, EF1α sequences also demonstrated high intra-isolate variation in *Pleurotus*. Even though at least 30 clones of each isolate were sequenced, genetic variation was not sampled exhaustively. Additionally, polymorphisms counts were probably underestimated since the quality checks were extremely stringent in the present study, and the actual heterogeneity level was likely to be much higher. For another protein-coding gene, *BiP*, Kuhn et al. [31] reported that sequence divergence in a single isolate of *Glomus irregulare* could be as high as 8%. Such high variation within individuals will influence the results of identification and phylogenetic analyses. We also detected inter- and intraspecific divergence of RPB2 and EF1α in the tested species. Results indicated that polymorphic sites of different isolates within 1 species could be different. Furthermore, divergence of RPB2 within *P. ostreatus* could be identical to that between *P. ostreatus* and *P. pulmonarius*, and intraspecific heterogeneity of EF1α in *P. ostreatus* could surpass the interspecific variability between *P. ostreatus* and *P. pulmonarius*.

These results suggested that high intra-isolate polymorphisms of RPB2 and EF1α are also likely widespread in other fungal groups, and interspecific variation might be less than or equal to intraspecific variation. This posed challenges to the molecular identification and phylogenetic studies on fungi. As we know, an ideal molecular marker used in molecular identification and phylogenetic studies is homogeneous within species, and interspecific variation exceeds intraspecific variation [1]. It could be speculated that the RPB2 and EF1α are too polymorphic to use for identification and phylogenetic studies in *Pleurotus* at the species level, and this hold true for other fungal groups. Ideally, these genes could be used for specific identification or phylogeny analyses only when high intra-isolate variation level is excluded by sequencing at least 10 clones of each sample. As already pointed out by Álvarez and Wendel [32], relying only on direct sequencing of a single PCR product may yield incorrect results due to the numerical inequality among several repeat types within one genome and the possible preferential amplification of one type. In the study of Lindner and Banik [8], they also indicated that phylogenetic analyses with the cloned sequences produced different trees relative to analyses with consensus sequences, and cloned sequences of a single isolate felled into more than one species clades or entirely new clades. If analyzed on their own, these cloned sequences of a single isolate most likely would be recognized as different “undescribed” or “novel” taxa [8]. However, screening for intra-isolate polymorphisms is absent in the routine use of RPB2 and EF1α in phylogenetic studies of fungal groups. Under such circumstances, specific divergence might have been underestimated in some groups, while species diversity overestimated.

Among the variants observed in RPB2 and EF1α, most were synonymous and would not cause amino acid change. On the other hand, very few indel polymorphisms were observed, and they were located in the intron region. This was largely due tomissense variations and indels that affected translation products were tended to be flushed out by natural selection. Of all detected variants, transitions between T and C showed the highest frequency. Additionally, most detected variants were single nucleotide polymorphisms, and there were some differences between isolates. These differences could probably be used to discriminate different isolates.

The reasons for intra-isolate polymorphisms could be manifold. Taq polymerase misreading is a potential contribution to sequence polymorphisms. In the present study, a repeated PCR with *pfu* polymerase and cloning was performed to evaluate the frequency of Taq misreadings. Results showed that Taq polymerase errors were in a very low rate or absent. Sequence deviation of the clone sequences of ITS, RPB2, and EF1α from different *P. ostreatus* isolates, as well as from *P. pulmonarius* and *P. citrinopileatus* isolates, were far out of the range of a misincorporation error of Taq polymerase. Additionally, we presumed that the polymorphisms occurring only in 1 of the 30 clones were Taq misreadings, and these sites were corrected. Therefore, Taq polymerase errors should not be considered as the source of the variations reported in our study.

The protein-coding genes RPB2 and EF1α usually present as single copy genes in fungi; however, high intraisolate polymorphism levels indicated that this is likely not always the case in *Pleurotus*. The RPB2 gene has been found to be multicopy in certain taxa in plants and trypanosomes [33], and multiple copies of EF1α have been reported in humans and in the spider genus *Habronattus* [34–36]. Multicopy genes are assumed to evolve under the mechanism of concerted evolution to maintain homogeneity of all copies [37]. However, intra-isolate sequence variability will accumulate if the conversion rate is lower than the rate of mutation, or if concerted evolution is slower than speciation. It is also possible that differences between mutation rates and DNA repair rates lead to an accumulation of sequence variants in genes with multiple copies [38]. As we know, heterokaryosis is present in fungi, and genetically differentiated nuclei might cause intra-isolate variation. However, the internal mechanism behind why rDNA and protein-coding genes show variants within single isolates in *Pleurotus* is still unclear and must be addressed in further research.

## Conclusions

In conclusion, we reported the polymorphisms of rDNA sequences and protein-coding genes of the tested species, which may also be present in other fungal groups. When inter- or intra-isolate variations of these markers are high, molecular identification or phylogeny analyses might cause opposing results. Especially when intra-isolate or intraspecific variation exceeds that of interspecific variation, individuals within 1 species might be considered as different species based on sequence comparisons. Consequently, identification and quantification of intra-isolate and intraspecific heterogeneity is of real importance, and more extensive sampling of these genes may be required to ensure reliability as desirable phylogenetic markers and DNA barcodes for specific identification.

## Additional files

Additional file 1: Figure S1. ITS polymorphic sites within *P. ostreatus*. Variable sites of 9 *P. ostreatus* are shown; polymorphisms could occur in the same sites, or different sites. (PDF 350 kb)

Additional file 2: Figure S2. Twenty-two ITS types observed within *P. ostreatus*. Two hundred and eighty-nine sequences of 10 *P. ostreatus* isolates were detected. (PDF 393 kb)

Additional file 3:Figure S3. Thirteen ITS types detected within a single isolate of *P. ostreatus* (isolate P021). (PDF 247 kb)

Additional file 4: Figure S4. Polymorphic sites of ITS sequences in the 3 *P. pulmonarius* isolates. Polymorphisms differed between isolates. (PDF 162 kb)

Additional file 5: Figure S5. ITS polymorphic sites in *P. citrinopileatus* isolates. No intra-isolate variant was detected, and 1 nucleotide substitution was observed between the 3 isolates. (PDF 68 kb)

Additional file 6: Figure S6. Polymorphisms of RPB2 sequences in *P. ostreatus* isolates. Variation of RPB2 within individuals in *P. ostreatus* was unexpectedly high. (PDF 1362 kb)

Additional file 7: Figure S7. Variants of RPB2 sequences in *P. pulmonarius* isolates. Indels were located in the intron region. (PDF 317 kb)

Additional file 8: Figure S8. Polymorphisms of RPB2 sequences in *P. citrinopileatus* isolates. Intra-isolate variations were found in the 3 isolates. (PDF 155 kb)

Additional file 9: Figure S9. Polymorphic sites of EF1α sequences in *P. ostreatus* isolates. Variation levels were differed markedly between isolates. (PDF 1487 kb)

Additional file 10: Figure S10. Variants of EF1α sequences in *P. pulmonarius* isolates. T/C transitions had the highest frequency. (PDF 278 kb)

Additional file 11: Figure S11. Sequence polymorphisms of EF1α in *P. citrinopileatus* isolates. Intra-isolate variation was observed only in isolate P145. (PDF 94 kb)

## Abbreviations

EF1α: elongation factor 1-alpha
ITS: Internal transcribed spacer
PCR: Polymerase chain reaction
rDNA: Ribosomal deoxyribonucleic acid
RPB2: RNA polymerase II second largest subunit
