# Supplementary material for: Intra- and inter-isolate variation of ribosomal and protein-coding genes in Pleurotus: implications for molecular identification and phylogeny on fungal groups
Source: BMC Microbiol. 2017 Jun 26;17:139. doi: 10.1186/s12866-017-1046-y (PMC5485676; doi:10.1186/s12866-017-1046-y)
Supplement: Supplementary file 1 — ITS polymorphic sites within P. ostreatus. Variable sites of 9 P. ostreatus are shown; polymorphisms could occur in the same sites, or different sites. (PDF 350 kb) [file 12866_2017_1046_MOESM1_ESM.pdf]

| Strains / Sites | 102 | 123 | 151 | 170 | 220 | 414 | 415 | 556 |
|---|---|---|---|---|---|---|---|---|
| P021 | T/C | T/– | T/A | A/– | T/A | C/– | T/– | T/C |
| P024 | T/C | T/– | T/A | A/– | T/A | C/– | T/– | T/C |
| P026 | T/C | T/– | T/A | A/– | T/A | C/– | T/– | T/C |
| P027 | T | T | A | A | A | T/– | T | C |
| P055 | T/C | T/– | T/A | A/– | T/A | C/– | T/– | T/C |
| P057 | T/C | T/– | T/A | A/– | T/A | C/– | T/– | T/C |
| P058 | T/C | T/– | T/A | A/– | T/A | C/– | T/– | T/C |
| P069 | T/C | T/– | T/A | A/– | T/A | C/– | T/– | T/C |
| P079 | T/C | T/– | T/A | A/– | T/A | C/– | T/– | T/C |
| P082 | T/C | T/– | T/A | A/– | T/A | C/– | T/– | T/C |
