# Supplementary material for: Intra- and inter-isolate variation of ribosomal and protein-coding genes in Pleurotus: implications for molecular identification and phylogeny on fungal groups
Source: BMC Microbiol. 2017 Jun 26;17:139. doi: 10.1186/s12866-017-1046-y (PMC5485676; doi:10.1186/s12866-017-1046-y)
Supplement: Supplementary file 2 — Twenty-two ITS types observed within P. ostreatus. Two hundred and eighty-nine sequences of 10 P. ostreatus isolates were detected. (PDF 393 kb) [file 12866_2017_1046_MOESM2_ESM.pdf]

Types / Sites	102	123	151	170	220	414	415	556
1	T	T	A	A	A	–	–	T
2	T	T	A	A	A	–	T	T
3	T	T	A	A	A	C	T	C
4	T	T	A	A	A	C	T	T
5	T	T	A	A	A	–	–	C
6	T	T	A	A	A	–	T	C
7	T	T	A	A	T	–	–	T
8	T	–	T	–	A	–	T	C
9	T	T	A	A	T	C	T	C
10	C	–	T	–	T	–	T	C
11	C	–	T	–	T	–	–	T
12	C	–	T	A	T	–	T	C
13	C	–	T	A	A	C	T	C
14	C	–	A	A	A	–	T	C
15	C	–	T	–	T	C	T	C
16	C	–	T	–	A	C	T	C
17	C	–	T	–	A	–	–	C
18	C	–	A	A	A	–	–	T
19	C	–	T	A	A	–	–	T
20	C	–	T	–	A	–	T	C
21	C	T	A	–	T	C	T	T
22	C	T	A	A	A	C	T	C
