# Supplementary material for: Intra- and inter-isolate variation of ribosomal and protein-coding genes in Pleurotus: implications for molecular identification and phylogeny on fungal groups
Source: BMC Microbiol. 2017 Jun 26;17:139. doi: 10.1186/s12866-017-1046-y (PMC5485676; doi:10.1186/s12866-017-1046-y)
Supplement: Supplementary file 3 — Thirteen ITS types detected within a single isolate of P. ostreatus (isolate P021). (PDF 247 kb) [file 12866_2017_1046_MOESM3_ESM.pdf]

Types / Sites	102	123	151	170	220	414	415	556
1	T	T	A	A	A	–	–	C
2	T	T	A	A	A	–	–	T
3	T	T	A	–	T	C	T	C
4	T	T	A	A	T	–	–	T
5	T	T	A	A	A	C	T	C
6	T	–	T	–	T	C	T	T
7	C	–	T	–	T	–	–	C
8	C	–	T	–	T	–	–	T
9	C	–	T	–	T	C	T	C
10	C	–	T	–	A	–	–	T
11	C	–	T	A	A	–	–	C
12	C	–	T	A	A	–	–	T
13	C	–	T	–	A	–	–	T
