# Supplementary material for: Intra- and inter-isolate variation of ribosomal and protein-coding genes in Pleurotus: implications for molecular identification and phylogeny on fungal groups
Source: BMC Microbiol. 2017 Jun 26;17:139. doi: 10.1186/s12866-017-1046-y (PMC5485676; doi:10.1186/s12866-017-1046-y)
Supplement: Supplementary file 4 — Polymorphic sites of ITS sequences in the 3 P. pulmonarius isolates. Polymorphisms differed between isolates. (PDF 162 kb) [file 12866_2017_1046_MOESM4_ESM.pdf]

Strains / Sites	87	186	213	306	413	417	450	484	531	572
P038	T/C	T	T/C	T/C	T	G/A	A	T/C	C	A
P041	T	T/C	T	T	T	A	A	C	C	T/A
P073	T	T	T	T	T/C	A	G/A	C	T/C	T
