# Supplementary material for: Intra- and inter-isolate variation of ribosomal and protein-coding genes in Pleurotus: implications for molecular identification and phylogeny on fungal groups
Source: BMC Microbiol. 2017 Jun 26;17:139. doi: 10.1186/s12866-017-1046-y (PMC5485676; doi:10.1186/s12866-017-1046-y)
Supplement: Supplementary file 5 — ITS polymorphic sites in P. citrinopileatus isolates. No intra-isolate variant was detected, and 1 nucleotide substitution was observed between the 3 isolates. (PDF 68 kb) [file 12866_2017_1046_MOESM5_ESM.pdf]

Strains / Sites	581
P145	-
P146	T
P147	T
