# Supplementary material for: Intra- and inter-isolate variation of ribosomal and protein-coding genes in Pleurotus: implications for molecular identification and phylogeny on fungal groups
Source: BMC Microbiol. 2017 Jun 26;17:139. doi: 10.1186/s12866-017-1046-y (PMC5485676; doi:10.1186/s12866-017-1046-y)
Supplement: Supplementary file 6 — Polymorphisms of RPB2 sequences in P. ostreatus isolates. Variation of RPB2 within individuals in P. ostreatus was unexpectedly high. (PDF 1362 kb) [file 12866_2017_1046_MOESM6_ESM.pdf]

Strains / Sites	9	15	16	17	18	19	21	24	28	61	82	83	121	127	190	193	196	202	214	220	226	245	250	286	298
P019	C	A	A	T	T/C	G	A	A	G	G	C	T/C	A	T	G	G	C	C	T	A	C/A	A	T/C	A	T
P027	G/C	C	G/A	T	T/C	G/A	G	G	G	G	C/A	T/C	A	T/C	G	G	C	C	T	A	C	A	C	G	T
P028	C	C/A	A	T/C	T	G	G	C/A	G/A	G	C/A	T	G/A	T/C	G/A	G/A	C/A	C/A	T/G	T/A	A	G/A	T/C	A	T/C
P053	C	C/A	A	T/C	T	G	G	C/A	G/A	G/A	C/A	T	G/A	T/C	G/A	G/A	C/A	C/A	T/G	T/A	A	A	T/C	A	T/C

Strains / Sites	319	322	325	328	331	337	352	364	367	376	379	394	397	400	412	415	421	467	480	486	490	493	508	523	526	532
P019	G	A	A	C	C	A	A	C	T	G	C	T	A	C	C	G	G/A	G/A	A	T	C	T	T/C	G	T	C
P027	G	A	A	C/A	C	A	C	C	T	G	T	C	A	T	A	A	A	A	A	T/C	C	T	T	G	T	C
P028	T/G	G/A	G/A	C	T/C	T/A	G/A	T/C	T/C	G/A	C	T/C	C/A	T	T/A	G/A	G	A	A	T	T/C	T/C	T/C	G/A	T/C	G/C
P053	T/G	G/A	G/A	C	T/C	T/A	G/A	T/C	T/C	G/A	C	T/C	C/A	T	T/A	G/A	G	A	G/A	T	T/C	T	T/C	G/A	G/T/C	T/C

Strains / Sites	538	569	571	577	589	592	616	617	628	640	646	649	653	661	664	670	677	679	682	683	687	697	709	721	730
P019	G	A	T/C	T/C	C	T/G	T	C	C	T/A	C	A	T/C	C/A	C	C	T/C	G	C	T/C	G/A	T	C	T	G
P027	G	G/A	C	C	C	G	T	C	C	T	C	A	C	C	C	C	T	G	C	T	A	T	C	T	G
P028	A	A	C	T/C	T/C	G	T/C	A	C	T/A	C	A	T/C	C/A	T/C	T/C	C	G/A	T/G	T/C	A	T/C	T/C	T/C	G/A
P053	A	A	C	T/C	C	G	T/C	A	T/C	T/A	T/C	G/A	T/C	C/A	C	C	T/C	G	T/C	C	A	T/C	T/C	T	G

Strains / Sites	751	754	784	799	805	808	826	828	835	841	847	859	862	883	886	904	910	922	928	940	955	958	961	994	1000
P019	T/C	A	A	G	G	T/G	G	A	A	C	A	T/C	C	C	A	T/C	G	C	G	T	C	G	T	C	G
P027	T	A	A	G	G	T	G	A	A	C	A	T	T	C	A	T	G	A	G	T	T	A	C	G	A
P028	C	T/A	A	G/A	A	T	G	A	G/A	T/C	G/A	T	T	G	G/A	T/C	G/A	C	G/A	T/A	C	G/A	T	C	G
P053	C	A	G/A	G/A	G/A	T	G/A	G/A	G/A	T/C	G/A	T	T	T	G/A	T/C	G/A	C	G/A	T/A	C	G/A	T	C	G
