# Supplementary material for: Intra- and inter-isolate variation of ribosomal and protein-coding genes in Pleurotus: implications for molecular identification and phylogeny on fungal groups
Source: BMC Microbiol. 2017 Jun 26;17:139. doi: 10.1186/s12866-017-1046-y (PMC5485676; doi:10.1186/s12866-017-1046-y)
Supplement: Supplementary file 7 — Variants of RPB2 sequences in P. pulmonarius isolates. Indels were located in the intron region. (PDF 317 kb) [file 12866_2017_1046_MOESM7_ESM.pdf]

| Strains / Sites | 8 | 9 | 10 | 11 | 18 | 19 | 20 | 45 | 200 | 230 | 275 | 284 | 291 | 302 | 320 | 350 | 378 | 416 | 492 | 566 | 593 | 712 | 830 | 848 | 908 | 917 |
|---|---|---|---|---|---|---|---|---|---|---|---|---|---|---|---|---|---|---|---|---|---|---|---|---|---|---|
| P003 | - | - | - | - | T | C | A | T | C | A | T | C | T/C | C | G/A | T/C | T | T/C | G/A | G/A | C | T/A | G/A | C | C | A |
| P077 | A/- | C/- | T/- | T/- | T/C | T/C | G/A | T/G | T/C | G/A | T/C | T/C | T/C | T/C | A | T/C | T/C | T | G | A | C | T | G | T | C | A |
| P078 | A/- | C/- | T/- | T/- | T/C | T/C | G/A | T/G | T/C | G/A | T | T/C | T/C | T/C | A | T/C | T/C | T | G | A | C/A | T | G | T/C | C/A | G/A |
