# Supplementary material for: Intra- and inter-isolate variation of ribosomal and protein-coding genes in Pleurotus: implications for molecular identification and phylogeny on fungal groups
Source: BMC Microbiol. 2017 Jun 26;17:139. doi: 10.1186/s12866-017-1046-y (PMC5485676; doi:10.1186/s12866-017-1046-y)
Supplement: Supplementary file 8 — Polymorphisms of RPB2 sequences in P. citrinopileatus isolates. Intra-isolate variations were found in the 3 isolates. (PDF 155 kb) [file 12866_2017_1046_MOESM8_ESM.pdf]

Strains / Sites	15	44	105	108	467	686	848	864
P145	T/C	T/C	T/C	G/A	T	A	T	T/C
P146	T/C	T	T/C	G/A	T	A	T	T/C
P147	T/C	T	T/C	G/A	T/C	G/A	T/C	T/C
