# Supplementary material for: Intra- and inter-isolate variation of ribosomal and protein-coding genes in Pleurotus: implications for molecular identification and phylogeny on fungal groups
Source: BMC Microbiol. 2017 Jun 26;17:139. doi: 10.1186/s12866-017-1046-y (PMC5485676; doi:10.1186/s12866-017-1046-y)
Supplement: Supplementary file 9 — Polymorphic sites of EF1α sequences in P. ostreatus isolates. Variation levels were differed markedly between isolates. (PDF 1487 kb) [file 12866_2017_1046_MOESM9_ESM.pdf]

Strains \ Sites	48	165	174	187	202	207	211	212	214	221	223	224	227	230	236	237	240	241	326	329	335
P019	T/C	T/C	C	A	T/A	T/A	T/C	T	T/G	T/C	C	C	A	C	A	T	T	T/C	T/C	T/C	T/C
P027	C	C	C	A/C	T	A	T	T/C	T	C	C	C	A	C	T/A	T	T	C	T	C	C
P053	C	T	T/C	A	T	T	T	T	G	C	G/C	G/C	G/A	C/-	T/A	T/C	T/G	C	T	T/C	G/C

Strains \ Sites	359	389	390	391	393	395	396	397	398	400	405	414	415	416	417	439	464	474	537	549
P019	C	G/A	T/C	C/A	G/A	A/-	T/-	G/-	A/-	T/C	T/C	G/A	G/A	T/C	G	C	A	T/C	C/A	T/C
P027	C	A	C	C	A	A	T	G	A	T	T/C	A	A	T	G	C	G/A	C	A	C
P053	T/C	A	T/C	C	G/A	A	T	G	A	T	T	A	A	T	G/A	C/A	A	C	A	C
