# Supplementary material for: Intra- and inter-isolate variation of ribosomal and protein-coding genes in Pleurotus: implications for molecular identification and phylogeny on fungal groups
Source: BMC Microbiol. 2017 Jun 26;17:139. doi: 10.1186/s12866-017-1046-y (PMC5485676; doi:10.1186/s12866-017-1046-y)
Supplement: Supplementary file 10 — Variants of EF1α sequences in P. pulmonarius isolates. T/C transitions had the highest frequency. (PDF 278 kb) [file 12866_2017_1046_MOESM10_ESM.pdf]

Strains / Sites	27	45	48	105	141	150	203	204	206	211	215	217	352	399	400	401	402	413	548
P003	T	T	T/C	T	T	T/C	T/C	G	A/T	A/G	T	G	T/C	C	G	A/C	T	A	T/C
P077	T/C	T	T	T/C	T/C	C	C	C/G	A	A	T/C	A/G	C	G/-	G/-	A/-	T/-	A/G	C
P078	T/C	T/C	T	T/C	T/C	C	C	C/G	A	A	T/C	A/G	C	G/-	G/-	A/-	T/-	A/G	C
