# Supplementary material for: Intra- and inter-isolate variation of ribosomal and protein-coding genes in Pleurotus: implications for molecular identification and phylogeny on fungal groups
Source: BMC Microbiol. 2017 Jun 26;17:139. doi: 10.1186/s12866-017-1046-y (PMC5485676; doi:10.1186/s12866-017-1046-y)
Supplement: Supplementary file 11 — Sequence polymorphisms of EF1α in P. citrinopileatus isolates. Intra-isolate variation was observed only in isolate P145. (PDF 94 kb) [file 12866_2017_1046_MOESM11_ESM.pdf]

Strains / Sites	189	214	223
P145	T/C	C	T/C
P146	C	A	T
P147	C	A	T
